# MPP1 Determines the Mobility of Flotillins and Controls the Confinement of Raft-Associated Molecules

**DOI:** 10.3390/cells11030311

**Published:** 2022-01-18

**Authors:** Agnieszka Biernatowska, Karolina Wójtowicz, Tomasz Trombik, Aleksander F. Sikorski, Aleksander Czogalla

**Affiliations:** 1 Department of Cytobiochemistry, Faculty of Biotechnology, University of Wrocław, ul. F. Joliot-Curie 14a, 50-383 Wrocław, Poland; 2 Department of Biotransformation, Faculty of Biotechnology, University of Wrocław, ul. F. Joliot-Curie 14a, 50-383 Wrocław, Poland; karolina.wojtowicz@uwr.edu.pl; 3 Department of Biophysics, Faculty of Biotechnology, University of Wrocław, ul. F. Joliot-Curie 14a, 50-383 Wrocław, Poland; tomasz.trombik@uwr.edu.pl; 4 Research and Development Center, Regional Specialist Hospital, Kamieńskiego 73a, 51-154 Wrocław, Poland; aleksander.sikorski@wssk.wroc.pl

**Keywords:** MPP1, flotillins, raft nanodomains

## Abstract

MPP1 (membrane palmitoylated protein 1) belongs to the MAGUK (membrane-associated guanylate kinase homologs) scaffolding protein family. These proteins organize molecules into complexes, thereby maintaining the structural heterogeneity of the plasma membrane (PM). Our previous results indicated that direct, high-affinity interactions between MPP1 and flotillins (raft marker proteins) display dominant PM-modulating capacity in erythroid cells. In this study, with high-resolution structured illuminated imaging, we investigated how these complexes are organized within erythroid cells on the nanometer scale. Furthermore, using other spectroscopic techniques, namely fluorescence recovery after photobleaching (FRAP) and spot-variation fluorescence correlation spectroscopy (svFCS), we revealed that MPP1 acts as a key raft-capturing molecule, regulating temporal immobilization of flotillin-based nanoclusters, and controls local concentration and confinement of sphingomyelin and Thy-1 in raft nanodomains. Our data enabled us to uncover molecular principles governing the key involvement of MPP1-flotillin complexes in the dynamic nanoscale organization of PM of erythroid cells.

## 1. Introduction

According to well-established knowledge, PM consists of many coexisting domains, heterogeneous and dynamic in nature and size (from nano- to microdomains), whose composition varies among cell types and determines their sophisticated functions. Raft nanodomains, referred to as dynamic sphingolipid- and sterol-dependent membrane assemblies, represent one of the major membrane organizing principles [1,2]. In living cells, raft nanodomains exist as small (˂100 nm), highly dynamic protein-lipid entities, formation of which is driven by capturing and stabilizing raft precursor nano-assemblies (nanoclusters) due to preferential interaction of protein and lipids. Upon signaling, raft nanodomains coalesce into larger micrometer domains (>200 nm) and act as sorting platforms, enabling the selective association of specific molecules to subsequently fulfill their role in numerous cellular processes [1,3,4]. Thus, from the perspective of the PM organization, raft nanodomains might be considered as functional, small building blocks, enabling the construction of larger microdomains and thus contributing to the spatiotemporal compartmentalization of the PM. In this particular context, raft nanodomain organization/formation has to be tightly coordinated. Due to the complexity of PM, this involves several overlapping processes, including spatial distribution and local accessibility of certain types of proteins and lipids, their dynamic cooperative co-clustering as well as the oligomeric nature of raft components. The outstanding question, then, is which biological factors might determine the self-organizing capacity and local stability of the existing raft nanodomains at the PM and whether such a mechanism(s) might occur as a general rule for different cell types.

MPP1, which belongs to the scaffolding MAGUK family, was recently identified by us as one of the candidates displaying raft-organizing capacity. We found that organization of functional raft nanodomains in erythroid cells is MPP1-dependent, as downregulation of MPP1 expression triggered significant changes in the lateral organization of the PM. This phenomenon was directly observed in the dramatic decrease in ordering parameters of cellular PM, meaningful loss of isolated DRM (detergent resistance membrane) fractions, which are considered to represent rafts biochemically [5], and changes in phase-separation properties of cell-derived GPMVs (giant plasma membrane vesicles) [6]. Indeed, the global MPP1-mediated PM alterations resulted in the loss of functional raft nanodomains, as raft-dependent receptor signaling pathways (insulin and c-kit receptors) were markedly affected, mainly at the level of H-Ras activity, in MPP1 knockdown cells [5,7]. In turn, our recent study emphasized that the molecular basis underlining such MPP1-driven raft nanodomain organization might be due to the direct interaction of MPP1 with the raft marker proteins flotillins [8,9]. Given the architecture of raft nanodomains, flotillins (flotillin 1 and 2) deserve special attention, as they play a crucial role in the arrangement of such domains [10,11]. These proteins are localized at the cytosolic leaflet of the PM of numerous cells, where they exist as homo- and hetero-tetramers of ~100 nm in size nanoclusters, stability of the latter being dependent on the presence of flotillin 2 [12,13]. Due to their oligomeric nature, mediated by the C-terminal flotillin domain, flotillin-based nanoclusters serve as a structural scaffold for raft nanodomains [14,15] and were shown to be implicated in the organization of specialized microdomains (>200 nm) involved in key important raft-mediated processes, e.g., in insulin signaling, GPI-protein endocytosis, and activation of T cells [16,17,18,19]. Flotillins, therefore, perfectly fulfill the criteria of being preferable MPP1-interacting partners in the context of raft nanodomain organization. Recently, we found that MPP1, via its central D5 domain, forms high-affinity (K_d_ ~30 nM) complexes with both flotillin 1 and 2 [9]. The organization of such complexes was revealed at the cytosolic face of the PM of erythroid cells and was confirmed in in vitro studies [8,9]. Of note, loss of the mutual, endogenous MPP1-flotillin interactions, due to the competitive overexpression of a peptide containing the fragment that binds flotillins or other competitors, affects both PM order and the insulin-raft-dependent signaling pathway in erythroid cells, emphasizing the physiological significance of these interactions in raft nanodomain organization [8,9]. Based on these data, we proposed a concept assuming the major role of MPP1 in promoting oligomerization of flotillin-based nanoclusters at the PM, which in turn stabilized them as functional raft nanodomains (also see Discussion). All these findings point our attention to an important aspect of the regulation of PM organization, where direct MPP1-flotillin interactions play an indispensable role in controlling the lateral PM heterogeneity, reflecting raft nanodomain organization.

This study, using state-of-the-art biophysical methods, including super-resolution structured illumination microscopy (SIM), demonstrated the nanoscale organization of MPP1-flotillin complexes in erythroid cells. Moreover, utilizing svFCS and FRAP, we revealed (in micro- and nanoscale) the precise in-depth picture containing molecular details concerning the impact of MPP1 on the dynamic behavior of flotillins and other raft-associated molecules. Our results show that MPP1 modulates the lateral mobility of flotillin 2 and determines the confinement of both sphingomyelin (SM) and Thy-1 protein in raft nanodomains of erythroid cells. Altogether, these data highlight that MPP1, as one of the fundamental biological factors that act as crucial raft-capturing molecules, is directly involved in the local stabilization and organization of raft nanodomains in living cells.

## 2. Materials and Methods

### 2.1. Cell Culture

Control (scrambled) and MPP1 knockdown HEL (MPP1 KnD) cells were obtained as described previously [5] and cultured in RPMI 1640 medium supplemented with 10% fetal calf serum, 2 mM glutamine, 2 µg/mL puromycin, 100 units/mL penicillin, and 100 μg/mL streptomycin at 37 °C in a humidified atmosphere of 5% CO_2_.

### 2.2. Plasmids

The Flot2-mEGFP plasmid was obtained by amplifying the flotillin 2 sequence (NM_004475.3; template: Flot_2_pcDNA/Hygro+ GenScript) in a PCR reaction (forward primer 5′ ATCTCGAGATGGGCAATTGCCACACGGTGGGGCCCAA 3′; reverse primer 5′ ATAAGCTTCACCTGCACACCAGTGGCCTTCTTGATCAGGGG 3′) and cloned into the mEGFPN1 plasmid (mEGFP-N1 was a gift from Michael Davidson; Addgene plasmid #54767; http://n2t.net/addgene:54767 (accessed on January 2021); RRID:Addgene_54767) using XhoI/HindIII restriction enzymes (New England Biolabs). The MPP1 “rescue” plasmid was obtained as described previously [6]. The eGFP-Thy-1 plasmid [20] was a kind gift of Dr. He and Dr. Marguet, CIML, Marseille.

### 2.3. Cell Transfection

Transient transfections of cells were performed by CLB (Lonza, Basel, Switzerland) electroporation according to the manufacturer’s protocol. For FRAP experiments, 2 × 10^6^ of control or MPP1 KnD cells were transfected with 1 µg of Flot2-mEGFP plasmids and analyzed after 24 h. For svFCS control, MPP1 KnD cells (2 × 10^6^) were transfected with 0.5 µg of eGFP-Thy-1 plasmids for 20 h. For MPP1 “rescue” expression, MPP1 KnD cells (2 × 10^6^) were transfected with 2 µg of MPP1 “rescue” plasmids for 48 h at 37 °C in a humidified atmosphere of 5% CO_2_.

### 2.4. SIM Microscopy

For SIM^2 analysis, 2 × 10^5^ cells were seeded onto poly-L-lysine coated coverslips and incubated for 30 min at 37 °C in a humidified atmosphere of 5% CO_2_. After incubation, cells were washed with PBS, fixed with 2% paraformaldehyde, and permeabilized with 0.1% Triton X-100. Next, cells were incubated for 30 min at room temperature (RT) with fetal bovine albumin (FBS) and then incubated overnight at 4 °C with antibodies directed against flotillin 2 (Abcam, Cambridge, UK; ab96507) or MPP1 (Abnova, Walnut, USA; H00004354- M01), followed by an additional 1 h incubation at RT with secondary antibodies labeled with Star Orange/Red fluorophore (Abberior, Gottingen, Germany). Coverslips were mounted on slides using the Abberior mount liquid antifade medium (Abberior). SIM^2 images were collected with a Zeiss ELYRA 7 with a Lattice SIM^2 super-resolution microscope (Carl Zeiss) using a 63x 1.4 NA oil immersion Plan-Apochromat objective lens at 30 °C. 561 nm and 642 nm laser lines were used to illuminate samples and an LBF 405/488/561/642 dichroic mirror was inserted into the optical path. Signals were collected by an sCMOS pco-edge 4.2M camera. Raw images (13 phase-shifted) were resolved by ZEN black (ZEN 3.0 SR FP2) software (Carl Zeiss, Jena, Germany) with the SIM^2 Lattice module. 

### 2.5. FRAP Experiments

For photobleaching experiments, 2 × 10^6^ control and MPP1 KnD cells were transiently transfected with Flot2-mEGFP, as described above. After 24 h, cells were washed with HBSS buffer (Gibco, Thermo Fisher Scientific, Amarillo, TX, USA) supplemented with 10 mM HEPES, pH 7.4 (HBSS/HEPES) and transferred onto poly-l-lysine coated Lab-Tek chambers and left for 20 min at 23 °C. For disruption of raft nanodomains, control cells transfected with Flot2-mEGFP were treated with 10 mM MßCD for 1 h at 37 °C, washed with HBSS/HEPES, and subsequently measured. All FRAP experiments were performed at 23 °C to minimalize polarization of flotillin 2 in the caps and only cells showing a uniform/homogeneous PM distribution of Flot2-mEGFP were taken for FRAP analysis. FRAP experiments were performed with a Zeiss LSM510 confocal scanning microscope (Jena, Germany). Images were acquired with a 40×, 1.2NA c-Apochromat water-immersion objective using a 488 nm argon laser as the excitation source. Argon laser power was set to its maximal value. Before bleaching, 5 pre-bleach frames were acquired using 3% transmission, and subsequently, photo-bleaching (5 iterations) of a circular region of interest (ROI) of 2.5 µm in diameter with 100% transmission of both 488 nm and 405 nm lasers was performed. Fluorescence recovery was monitored for an additional 100 frames at 3% transmission. Fluorescence recoveries during the time series and the half-time recovery (t_1/2_) and mobile fraction were quantified using Zeiss LSM510 software (ZEN 2007, Jena, Germany).

### 2.6. svFCS Measurements

The svFCS measurements were performed using a custom-made svFCS optical system based on a classical Axiovert 200 M fluorescence microscope (Carl Zeiss, Oberkochen, Germany) according to the protocol described by Mailfert et al. [21,22]. Briefly, the waist size was calibrated with 2 nM Rhodamine 6G solution and 488 nm laser beam illumination at the intensity of 330 µW. Living cells analyses were performed under physiological conditions at 37 °C and the cells were maintained in the acquisition medium (HBSS/HEPES buffer). The 488 nm laser beam was adjusted to 2–4 µW and the signal was collected by a series of 20 runs lasting for 5 s each. The measurements were carried out on 10 to 20 individual cells and the obtained data were analyzed by the IGOR Pro program. The collected autocorrelation functions were fitted with a 2D lateral diffusion model and the mean diffusion time $\tau_{d}$ was calculated. Four waists were analyzed in order to construct a single diffusion law.

### 2.7. Labeling with Fluorescence Lipid Analog

Before svFCS measurements, 5 × 10^5^ control and MPP1 KnD cells were labeled with SM-BODIPY or PC-BODIPY (Thermo Fisher Scientific, Waltham, MA, USA) (0.075 μM lipid analog/BSA complex) in HBSS/HEPES buffer for 10 min at RT. Cells were washed with HBSS/HEPES and transferred to poly-l-lysine coated Lab-Tek chambers, left to settle for 15 min at 37 °C, and subsequently measured. For metabolic inhibition of sterol and sphingolipid synthesis, 1 × 10^7^ control cells were treated with 25 µM zaragozic acid for 48 h in complete RPMI medium and then incubated for an additional 18 h in RPMI medium supplemented with 10% inactivated FBS (Biowest, Riverside, CA, USA), 25 µM zaragozic acid (ZA) and 10 µM myriocin (Myr) at 37 °C in a humidified atmosphere of 5% CO_2_ as previously described [23]. The total level of cholesterol in control and treated cell lysates was determined with Amplex Red Cholesterol Kit Assay (Thermo Fisher) (see Figure S6). After treatment, cells were labeled with SM-BODIPY and svFCS measurements were carried out as described above. 

### 2.8. Statistical Analysis

All statistical analyses were performed using GraphPad Prism 6.0. (GraphPad Software, San Diego, CA, USA).

## 3. Results

### 3.1. MPP1-Flotillin Complexes Exist as either Small Clusters or Large Polarized Preassembled Caps in the PM

**Figure 1:**
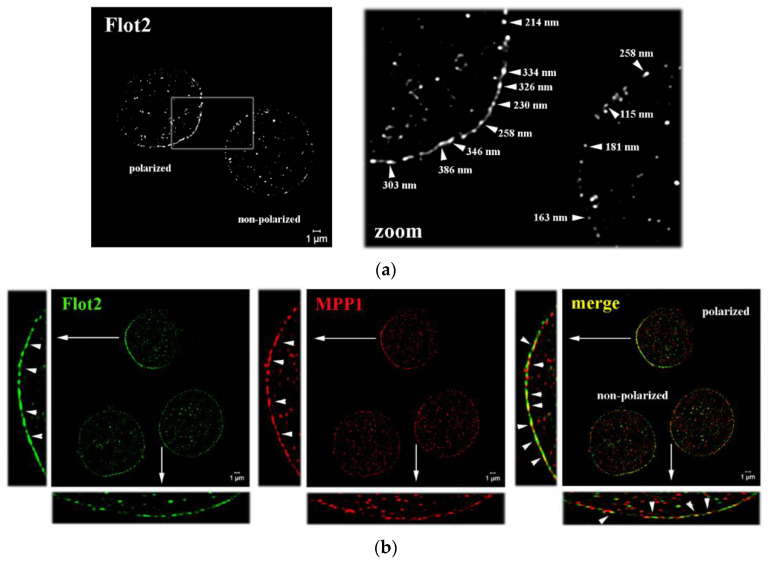
MPP1 forms both small nano- and large micrometer size complexes with flotillins in the PM of erythroid cells (**a**) Representative SIM^2 images showing organization of endogenous flotillin 2 (Flot2) in highly polarized preassembled caps (polarized cells) or small flotillin nanoclusters uniformly distributed along the PM (non-polarized cells) of control HEL cells (left). The blank rectangle corresponds to the zoomed region (right) of the selected area wheres the estimated sizes of flotillin complexes were indicated (also see Figure S1d). (**b**) Representative SIM^2 images showing co-localization of Flot2 and MPP1 in control HEL cells. Two types of MPP1-flotillin complexes are found, large polarized-micrometer size (in flotillin 2-caps) and small uniformly distributed nanometer-size clusters (in non-polarized cells). Size estimation is shown and described in Figure S3. Scale bar, 1 µm. Images represent the central z-stack.

Our recent research showed that high-affinity complexes of MPP1 and flotillins functionally control the lateral organization of PM in erythroid cells [8,9]. Here, by using SIM microscopy, we were able to visualize the organization of these complexes in the nanoscale range. We focused our imaging on flotillin 2, which, unlike flotillin 1, regulates the stability of hetero-tetramers [15] and is stably anchored to the PM by means of both myristylation and palmitoylation [14]. Surprisingly, we observed that the distribution of endogenous flotillin 2 is not homogeneous across the PM of resting HEL cells, and therefore two populations of cells have been characterized. In ~50% of cells, flotillin 2 was found organized in the preassembled caps and formed polarized regions in the PM (Figure 1a), organization of which was not dependent on MPP1 expression (see Figure S1). In these specific areas, flotillin 2 forms characteristic clusters ~200–400 nm in size, which are arranged in close proximity to each other and seem to locally assemble into larger ones, forming dense cap-like structures (see Figure S3). Such asymmetric localization of flotillins into preassembled platforms has previously been observed as a characteristic feature of some white blood cells, including lymphocytes, leukocytes, and neutrophils [19,24,25], but it has not been found in erythroid cells before. Interestingly, a similar “polarized” distribution pattern was observed for MPP1 (Figure 1b; see Figure S2). In flotillin 2-polarized cells, MPP1 exhibited striking cap-like localization, forming densely distributed assemblies. In this area, numerous spots of co-localization of MPP1 and flotillin 2 were visible. On the other hand, in the flotillin 2 non-polarized cells (Figure 1a) flotillin 2 forms point-like, small clusters (~100–200 nm) that are separated from each other and are uniformly distributed along the PM, which most likely correspond to the flotillin hetero-tetramers also observed in other cell types [12,13,15]. In these particular cells, MPP1 co-localizes pointwise with flotillin 2 in numerous regions along the PM, forming ~100–200 nm merged co-assemblies (Figure 1b; see also Figure S3). These interesting findings emphasize, therefore, that in erythroid cells, MPP1-flotillin complexes are organized in small clusters distributed along with the PM. However, under specific (still undefined) physiological condition(s), MPP1 is selectively recruited to the preassembled flotillin 2 caps, where both co-assemble and form visible, distinctive polarized platforms (microdomain assemblies).

### 3.2. Loss of MPP1 Increases the Lateral Mobility of Flotillin 2 at the PM

**Figure 2:**
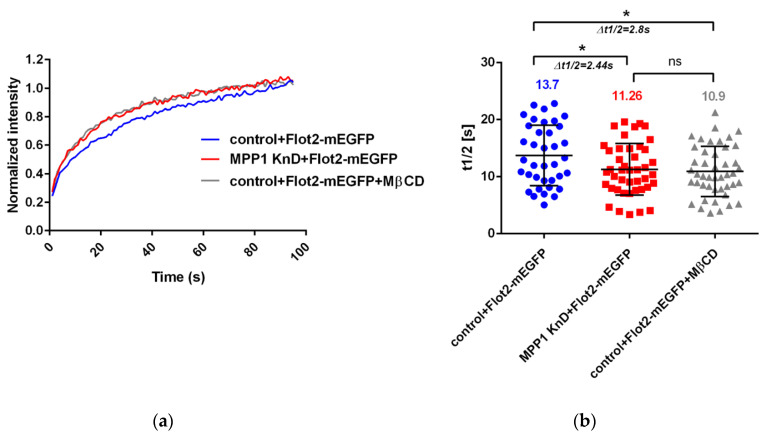
MPP1 limits the lateral mobility of flotillins at the PM. FRAP measurements showing (**a**) normalized average fluorescence recovery curve and (**b**) half-recovery time (t_1/2_) of the overexpressed Flot2-mEGFP in control (n = 37), MPP1 KnD cells (n = 43) and raft-disrupted MßCD-treated control cells (n = 45). Data represent three independent experiments. Statistical analysis was performed using non-parametric *t*-test *p* ˂ 0.05 (*), error bars correspond to the SD.

In order to gain detailed insight into the behavior of the MPP1-flotillin complexes at the PM, we used the FRAP technique to follow their molecular dynamics in living cells. Therefore, we overexpressed flotillin 2 (Flot2-mEGFP) and analyzed its lateral mobility in the control and MPP1 knockdown cells (MPP1 KnD). First, the optimal overexpression level of Flot2-mEGFP was chosen, to ensure a comparable level with endogenous protein (see Figure S4a). After transfection, over 50% of overexpressed Flot2-mEGFP was accumulated in caps in both control and MPP1 KnD cells, similarly to what was observed for endogenous protein (see Figure S4b,c). Since the mobility of flotillin 2 within the caps was previously described to be essentially immobile [19] for FRAP experiments, cells showing non-polarized, uniform distribution of Flot2-mEGFP along the PM were selected. As shown in Figure 2, we observed a significant increase in the lateral mobility of overexpressed Flot2-mEGFP in PM of MPP1 KnD cells. The fluorescence recovery half time was decreased by approximately ~2.44 s in MPP1 KnD cells (t_1/2_ = 11.26 ± 3.55 s) compared to the control cells (t_1/2_ = 13.7 ± 4.51 s). These alterations were not associated with a change in the total amount of mobile fraction of Flot2-mEGFP, which in both control (42.13% ± 4.01) and MPP1 KnD (41.74% ± 4.15) cells was comparable. Moreover, a similar increase in the lateral mobility of Flot2-mEGFP was observed after chemical disruption of raft nanodomains in MßCD-treated cells (t_1/2_ = 10.9 ± 3.49 s), where the mobility shift was approximately ~2.8 s, while maintaining ~42% (±3.97) of the mobile fraction, compared to the control cells. No statistically significant differences in half-time recovery of Flot2-mEGFP between MPP1 KnD and MßCD-treated cells were detected, therefore, indicating the crucial involvement of MPP1/cholesterol-dependent raft nanodomains in the regulation of the lateral diffusion of Flot2-mEGFP in the PM of erythroid cells.

### 3.3. MPP1 Determines the Molecular Diffusion and Raft Partitioning of Sphingomyelin

**Figure 3:**
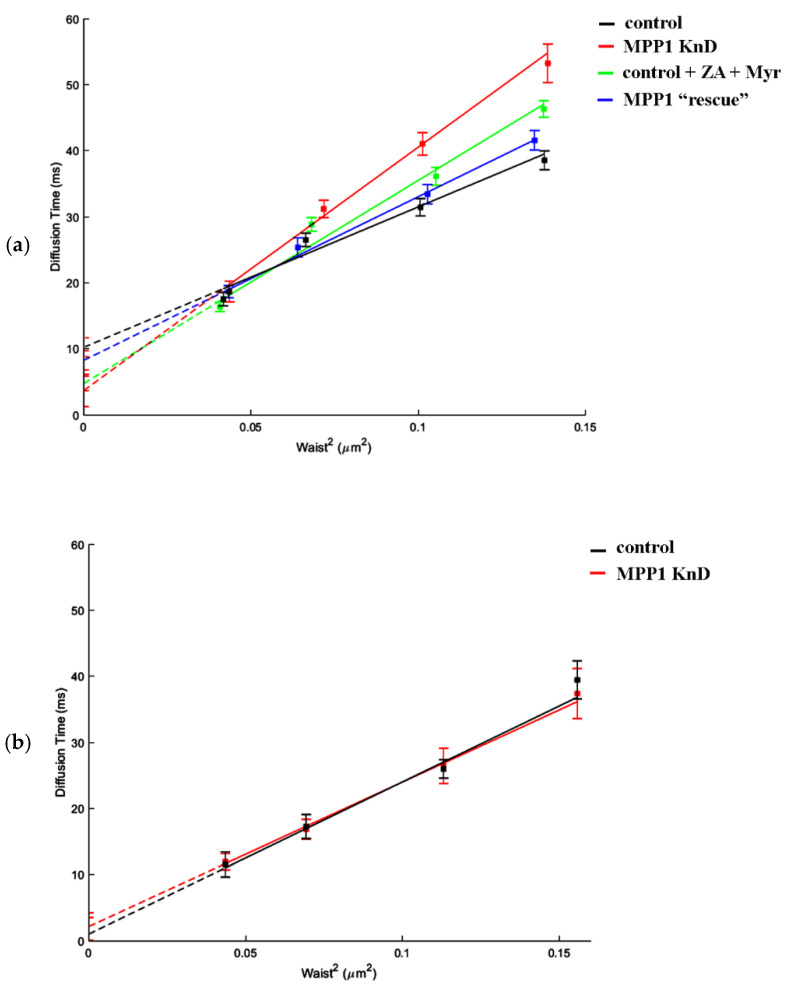
The switchable impact of MPP1 on the molecular mobility and confinement of SM in raft nanodomains. svFCS measurements and representative diffusion laws for (**a**) fluorescently labeled sphingomyelin (SM-BODIPY) and (**b**) phosphatidylcholine (PC-BODIPY) in HEL cells.

Next, to investigate how the downregulation of MPP1 impacts the processes occurring locally, we took advantage of svFCS, which allows one to precisely measure the diffusion parameters of molecules at the PM of living cells with high spatial-temporal resolution [21,22]. In this method, the diffusion time is analyzed as a function of the different size of the observed area (waist, ω), enabling the determination of a t_0_ value which estimates whether the diffusion of an analyzed molecule is dependent on temporal entrapment in membrane nanodomains (t_0_ > 0), is cytoskeleton-influenced (t_0_ ˂ 0) or represents unlimited free diffusion (t_0_ = 0) [21]. For this purpose, fluorescently labeled sphingomyelin (SM-BODIPY) was used as a specific sensor, reflecting dynamic changes in raft nanodomain organization. First, the diffusion parameters were measured in the PM of control cells, where the SM analog displayed a characteristic positive t_0_ value (t_0_ = 10.25 ± 1.43 ms), indicating that its lateral diffusion is highly dependent on partitioning in PM nanodomains (Figure 3a, black line; Table 1). Notably, we observed a significant, almost three-fold decrease in the confinement of SM in the PM of MPP1 KnD, where the t_0_ value dropped to 3.71 ms (Figure 3a; red line), thus resembling more unlimited, Brownian-like diffusion of SM. A similar “diffusion pattern” of SM was obtained after combined metabolic treatment of control cells with zaragozic acid (ZA) and myriocin (Myr), which are known to reduce the endogenous levels of SM and cholesterol, respectively, and affect the lipid raft nanodomains [23]. As shown in Figure 3a, the lateral diffusion of SM-BODIPY is certainly sensitive to destabilization of raft nanodomains, as indicated by the decrease in t_0_ value, which reached 4.77 ms (green line) in the PM of treated cells (also see Figure S6). The consistency of these results underlines that MPP1 controls the confinement of SM in raft nanodomains and thus determines the molecular dynamics and architecture of such domains. However, to verify this MPP1-dependent switching process, we measured the molecular diffusion of SM-BODIPY in MPP1 KnD cells in which the expression of MPP1 was transiently restored (MPP1 “rescue”; blue line; see also Figure S7), and observed almost complete recovery of the SM-BODIPY diffusion parameters, where the t_0_ value increased from 3.71 ms (MPP1 KnD) to 8.28 ms (MPP1 “rescue’), and the latter was comparable with the control cells (10.25 ms). Indeed, we found that MPP1 affects only the molecular diffusion of “raft-partitioning lipids”, as no significant changes in the diffusion parameters of fluorescently labeled phosphatidylcholine (PC-BODIPY), which represents the free diffusion mode at the PM, were observed in our svFCS analysis between control and MPP1 KnD cells (Figure 3b). 

### 3.4. MPP1 Contributes to Thy-1 Confinement in Raft Nanodomains

**Figure 4:**
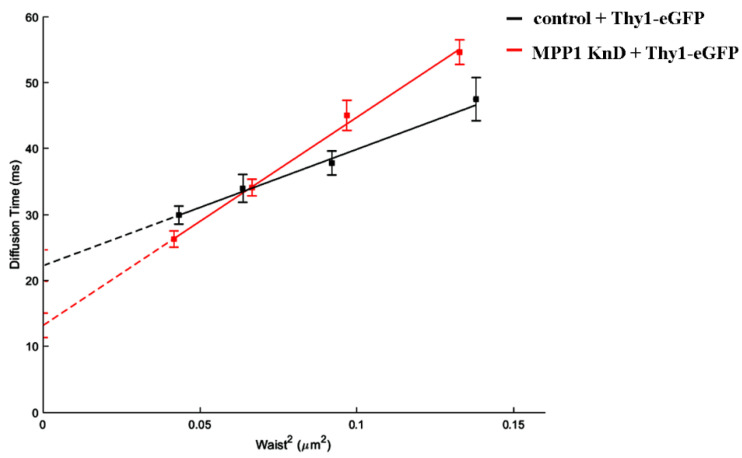
MPP1 affects the dynamics and confinement of Thy-1 in raft nanodomains. The diffusion law of eGFP-Thy-1 transfected control and MPP1 KnD cells.

To extend the molecular basis of the MPP1-dependent contribution in raft nanodomain organization, we subsequently examined the lateral diffusion of another raft-related molecule. For this, we choose GPI-anchored Thy-1 protein (eGFP-Thy-1), which was previously found to associate with flotillin 2 [12,13]. On the other hand, in contrast to flotillin 2, in our study Thy-1 was uniformly distributed at the PM of erythroid resting cells, thus allowing a homogeneous population of cells to be obtained for further evaluation in svFCS measurements. Our analysis showed that eGFP-Thy-1 exhibits constrained diffusion with a positive t_0_ value reaching ~22 ms at the PM of control cells (Figure 4; black line), which is in agreement with data observed by others [22,23,26]. Downregulation of MPP1 strongly impaired the confinement of Thy-1 in raft nanodomains, which is reflected by the drop of the observed t_0_ value approximately two-fold to ~13 ms (Figure 4; red line), illustrating that the effective raft-partitioning diffusion mode is determined by the presence of MPP1, thus indicating its crucial role in the molecular organization of raft nanodomains. 

## 4. Discussion

Determination of factors regulating the self-organization and stability of raft nanodomains is a key issue considering their crucial impact in spatiotemporal organization of PM and involvement in the regulation of numerous cellular processes, especially those related to cancer progression [27]. Our earlier studies indicated that the interplay between MPP1 and flotillins is critical for the lateral organization of the PM of erythroid cells, and suggested that mutual MPP1-flotillin interactions act as a major driving force for functional raft nanodomain organization [5,6,7,8,9]. Here, we wanted to reveal the molecular details underlying this process by examining how such complexes are organized at the nanoscale level and how MPP1 affects the dynamics of flotillins as well as other raft-associated molecules from both the global (in microscale) and local (in nanoscale) perspective. First, we found that in resting erythroid cells, flotillins display a similar distribution pattern as that observed previously for other hematopoietic cells [19,24,25] and exist in the PM either as small evenly distributed flotillin clusters (representing hetero-oligomers) or form large, preassembled polarized caps at one pole of the cell (Figure 1). Due to this fact, two major types of MPP1-flotillin complexes can be distinguished at the PM of erythroid cells. In cells exhibiting non-polarized flotillin distribution, MPP1-flotillin complexes exist as nanodomains, observable as point-like clusters, distributed throughout the whole PM. Considering their small size (~100–200 nm), these complexes most likely represent native raft nanodomains. On the other hand, in flotillin polarized cells, MPP1 seems to be selectively targeted into micro-size preassembled-caps, where it co-assembles with flotillins and thus co-exists in higher-structured, micrometer-sized domains. Although MPP1 is found in flotillin caps, its presence is not indispensable for such micro-scale domain organization (see Figure S1). Noticeable accumulation of MPP1 in these regions is, however, interesting. Several reports have shown that flotillin caps acts as priming platforms for the assembly of multiprotein complexes and thus recruit signaling molecules upon raft-dependent activation, contributing therefore to activation and polarization of immune cells [19,24,25]. Of note, MPP1 was found to be a critical regulator of neutrophil polarity upon chemiotactic simulation [28], while flotillins (flotillin caps) were shown to be directly implicated in the formation of the neutrophil uropod upon chemotaxis [29]. These facts thus emphasize that both flotillins and MPP1 are critical in structuring neutrophil polarity, although their mutual dependencies have not yet been defined. Based on these data, one might speculate that in erythroid cells, flotillin caps could play a similar role and serve as sequestering platforms, implicated in numerous processes linked with signaling or even differentiation of erythroid precursors during erythropoiesis. However, the exact role of flotillin caps in erythroid cells, the specific role MPP1 plays in these domains and the molecular basis that underlies its specific redistribution are still open questions that require further research.

**Figure 5:**
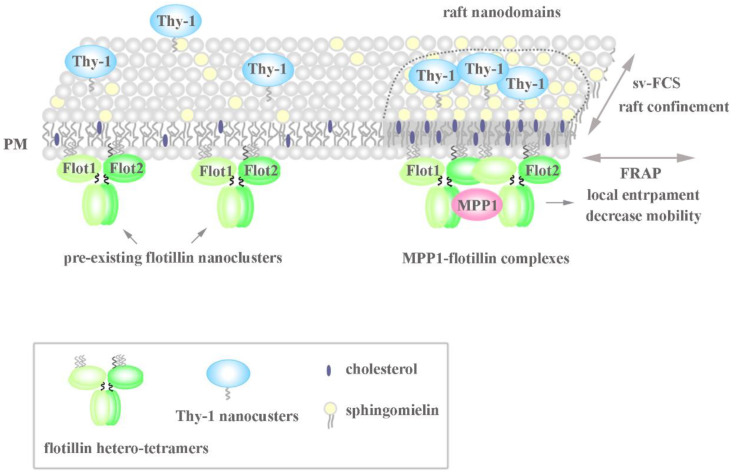
Model of the MPP1-flotillin dependent organization of raft nanodomains. MPP1 acts as a raft-sequestering molecule and organizes raft nanodomains due to mutual interactions with flotillins. The high-affinity binding of MPP1 to the pre-existing flotillin nanoclusters enhances their oligomerization and limits their lateral mobility in the PM, although the exact stoichiometry is not known. Such temporal immobilization triggers changes in the surrounding environment resulting in locally elicited concentration and confinement of other raft-associated molecules such as sphingomyelin and Thy-1. This rearmament drives the physicochemical changes in the PM, resembling the organization of more ordered domains reflecting functional raft nanodomains (for more details, see the text). MPP1-flotillin complexes act therefore as basic structural units organizing functional raft nanodomains.

Being focused on the biogenesis of raft nanodomains and factors responsible for their nanoscale organization and stabilization, in the next part of the study, we investigated cells where MPP1-flotillin complexes were organized in small, uniformly distributed nanodomains. Wondering how the presence of MPP1 impacts the lateral mobility of flotillins, we used FRAP and found that MPP1 has a capacity to reduce the dynamics of flotillins in the PM. Loss of MPP1 was associated with significant increases in Flot2-mEGFP mobility (~2.44 s shift) while having no impact on its mobile fraction. Similar Flot2-mEGFP behavior was observed when the organization of raft nanodomains was partially disrupted (~2.8 s shift), illustrating that flotillin dynamics is strongly controlled by its association with MPP1-dependent raft nanodomains. This was strengthened by the fact that the increased mobility of flotillins was not related to their enhanced redistribution between PM and internal membranes in MPP1 KnD cells (see Figure S5), indicating that these alterations are directly driven by the loss of interactions with MPP1, which acts as a molecular anchoring point. Based on these data, we concluded that binding of MPP1 to flotillin 2 (Flot2-mEGFP) sequesters flotillin nanoclusters, triggering local entrapment and reducing their lateral mobility. Indeed, our svFCS data revealed the switchable impact of MPP1 on the behavior and molecular dynamics of other raft-related molecules. We demonstrated that MPP1 affects the dynamics of raft lipid precursor/components such as sphingomyelin (SM-BODIPY), thus defining its confinement in raft nanodomains. This effect was reflected by a significant drop in the t_0_ value (three times lower for MPP1 KnD cells), thus giving a straightforward message of a largely unlimited, raft-independent diffusion mode of sphingomyelin analog upon deprivation of MPP1 from the PM. Downregulation of MPP1 triggered similar changes in molecular behavior of SM-BODIPY as those observed after treatment of control HEL cells with raft-disrupting factors shown here and also in other studies [23,26]. This emphasizes the high potential of MPP1 as a raft organizer. Of note, such an impact was not detected for the non-raft lipid analog, indicating the selective nature of MPP1-dependent local immobilization of raft lipids. Moreover, the reversible character of the mechanism shown here, by overexpression of the MPP1 “rescue” mutant, as well as the involvement of MPP1 in molecular dynamics and confinement of flotillin 2-associated Thy-1 protein in raft nanodomains, leaves no question that MPP1 acts as a critical tuning molecule in organizing and stabilizing local, lateral heterogeneity. Altogether, these observations help to identify a precise mechanism whereby the organization of functional raft nanodomains is strikingly controlled by endogenous factors such as MPP1. Our data point, therefore, to the novel concept of the involvement of MPP1 in capturing and stabilizing the pre-existing flotillin nanoclusters into functional raft nanodomains. In this view, the binding of MPP1 to flotillins enhances their oligomerization and co-assembly, thus inducing their local immobilization. This, in turn, triggers changes in the surrounding environment, resulting in temporal concentration and entrapment of sphingomyelin, or Thy-1 nanoclusters [30,31], and probably other raft-preferring molecules. Such specific MPP1-dependent rearrangement drives the physicochemical changes in the PM, resembling the organization of more ordered domains reflecting functional raft nanodomains (Figure 5). MPP1 acts, therefore, as an endogenous raft-sequestering molecule, switching the cooperative co-clustering of proteins and lipids. Our proposal is in line with others [3] and highlights the principle role of MPP1 in promoting oligomerization of raft-associated elements (flotillin nanoclusters) as a dominant driving force orchestrating PM raft organization. In this scenario, MPP1-flotillin complexes represent a basic organizational unit, selectively attracting and sequestering raft-related lipids, GPI nanoclusters, and other molecules, leading to local subcompartmentation of the PM. Such MPP1-driven immobilization of flotillin nanoclusters fits well with the view of Garcia-Parajo et al., assuming the functional remodeling of pre-existing nanoclusters as a major feature of PM organization [32]. Interestingly, a similar observation of protein-induced reorganization of PM properties was published recently by Urbancic et al., who found that partial immobilization of proteins such as the T-cell receptor elicits local membrane order and enhances interactions with raft-preferring molecules such as Lck in GPMVs [33]. These data are consistent with our previous observation of tunable changes in MPP1-derived phase separation behavior of GPMVs that were linked directly with the loss of MPP1 and were not associated with the alterations of major lipid composition [6]. This, in turn, illustrates that the temporal stabilization of protein nanoclusters, and hence their local entrapment, directly translates into changes in the local surroundings, indicating that the physicochemical properties of the PM are actively tunable by proteins.

In summary, our results provide new molecular details underlying the MPP1-flotillin-based mechanism of lateral PM organization, emphasizing the critical, switchable role of MPP1 in capturing and stabilizing pre-existing flotillin nanoclusters, thus enabling the spatial concentration and confinement of other raft-associated molecules into functional raft nanodomains. Assuming the ubiquitous expression of both flotillins [10,34] and MPP1/or MAGUK proteins [35,36], we propose that such mutual complexes act as key regulatory and structuring endogenous units controlling lateral PM organization in living cells. Unrevealing the molecular basis that governs the lateral PM heterogeneities is extremely important in the context of cancer progression and metastasis, where raft compartmentalization promotes receptor oligomerization and enhances signal transduction, therefore being a master regulator in cancer signaling [37]. Given the large and growing number of reports implicating overexpression of flotillins in the poor survival prognosis and high metastatic progression in a wide range of cancers [37,38], as well as data revealing the MPP1-related mechanism of drug resistance in leukemia [39], elucidation of the interplay between MPP1-flotillin and raft nanodomain organization might be helpful as a further potential therapeutic target.

## Figures and Tables

**Table 1:** Summary of the diffusion parameters assessed by svFCS.

**SM-BODIPY**	**T_0_ [ms]** **±** **SEM**	**D_eff_ [** **µ** **m^2^/s]** **±** **SEM**
control	10.25 ± 1.43	1.18 ± 0.09
MPP1 KnD	3.71 ± 2.44	0.68 ± 0.06
control + ZA + Myr	4.77 ± 1.08	0.81 ± 0.04
MPP1 ”rescue”	8.28 ± 1.46	1.01 ± 0.07
**PC-BODIPY**	**T_0_ [ms]** **±** **SEM**	**D_eff_ [** **µ** **m^2^/s]** **±** **SEM**
control	1.04 ± 2.49	1.09 ± 0.12
MPP1 KnD	2.18 ± 2.04	1.15 ± 0.15
**eGFP-Thy-1**	**T_0_ [ms]** **±** **SEM**	**D_eff_ [** **µ** **m^2^/s]** **±** **SEM**
control	22.26 ± 2.37	1.42 ± 0.26
MPP1 KnD	13.21 ± 1.85	0.79 ± 0.06

## Data Availability

Further information and requests for resources and reagents should be directed to and will be fulfilled by the Lead Contact, Aleksander Czogalla (aleksander.czogalla@uwr.edu.pl). Materials and plasmids generated in this study are available upon request from the Lead Contact.

## Supplementary Materials

The following are available online at https://www.mdpi.com/article/10.3390/cells11030311/s1, Figure S1: Flotillins are organized in small nanoclusters or in large preassembled caps in control and MPP1 KnD cells, Figure S2: MPP1 is selectively distributed to the flotillin 2 preassembled caps, Figure S3: Size measurements of the MPP1-flotillin 2 complexes in HEL cells, Figure S4: Analysis of overexpression level of Flot2-mEGFP in HEL cells, Figure S5: Analysis of the distribution of flotillins in the PM and internal membranes, Figure S6: The effect of metabolic treatment of ZA and Myr on the total cholesterol level in HEL cells, Figure S7: Restoration of MPP1 using MPP1 “rescue” overexpression in MPP1 KnD cells.
