# Supplementary material for: MPP1 Determines the Mobility of Flotillins and Controls the Confinement of Raft-Associated Molecules
Source: Cells. 2022 Jan 18;11(3):311. doi: 10.3390/cells11030311 (PMC8834348; doi:10.3390/cells11030311)
Supplement: Supplementary file 1 [file cells-11-00311-s001.zip › cells-1523240-supplementary.pdf]

Supplementary Materials

MPP1 determines the mobility of flotillins and controls the confinement of raft-associated molecules

Agnieszka Biernatowska[1]*, Karolina Wójtowicz[2], Tomasz Trombik[3], Aleksander F. Sikorski[4], Aleksander Czogalla[1]*

Supplementary Figure 1

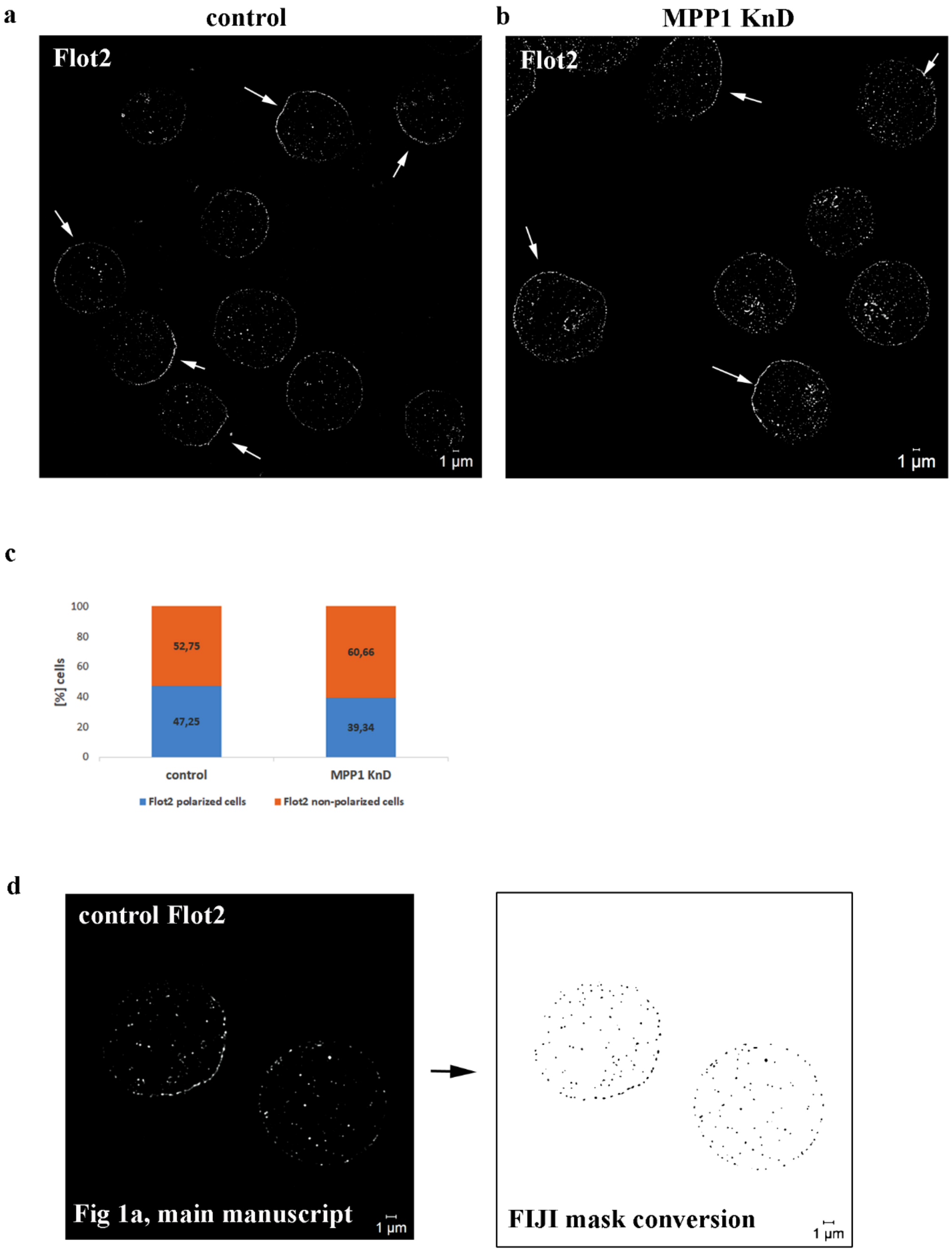

Figure S1. Flotillins are organized in small nanoclusters or in large preassembled caps in control and MPP1 KnD cells.

(a, b) SIM^2 images showing distribution of endogenous flotillin 2 (Flot2) in control and MPP1 KnD cells. In both cell types Flot2-caps are present (arrows) (c) Average percentage of flotillin 2-polarized and flotillin 2 non-polarized cells observed in the control and MPP1 KnD cells respectively. (d) FIJI software was used to measure the size of flotillin 2 nanoclusters presented in Fig. 1a (main manuscript) and here on the left. Briefly, the SIM^2 image was filtered with a Gaussian blur filter (sigma = 1) to reduce noise and then a Bernsen local threshold was applied to segment objects of interest. The radius of Bernsen local threshold was set to 15 whereas both parameters 1 and 2 were set to 0. Segmented regions were measured using the Analyse Particle function (the following settings were used: Size: 0 to infinity; Circularity: 0-1; objects on edges were excluded). The sizes of segmented spots (arrows in Figure 1a main manuscript) were expressed as Ferret's diameter.

Supplementary Figure 2

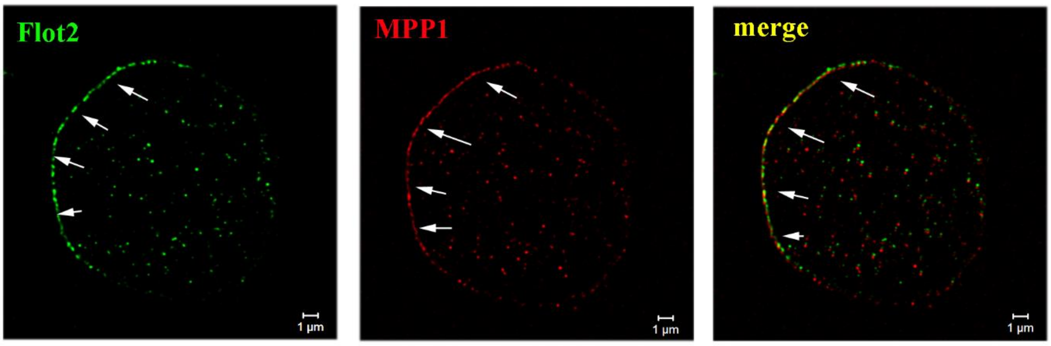

Figure S2. MPP1 is selectively distributed to the flotillin 2 preassembled caps.

SIM^2 images precisely showing selective accumulation of MPP1 in flotillin 2 preassembled caps. Arrows show proteins accumulated in caps. Scale bar, 1 µm.

Supplementary Figure 3

a

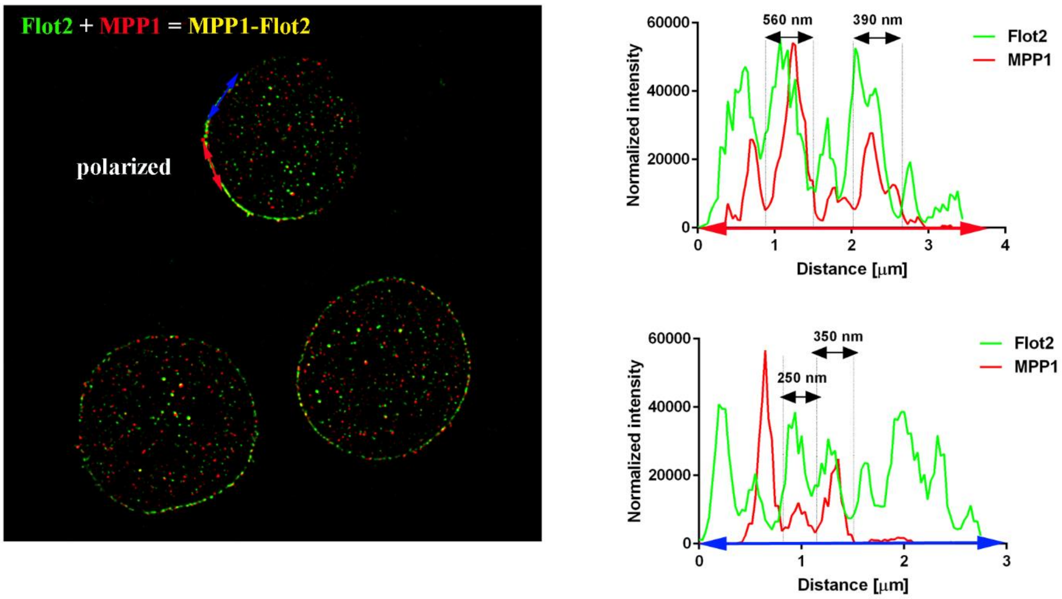

b

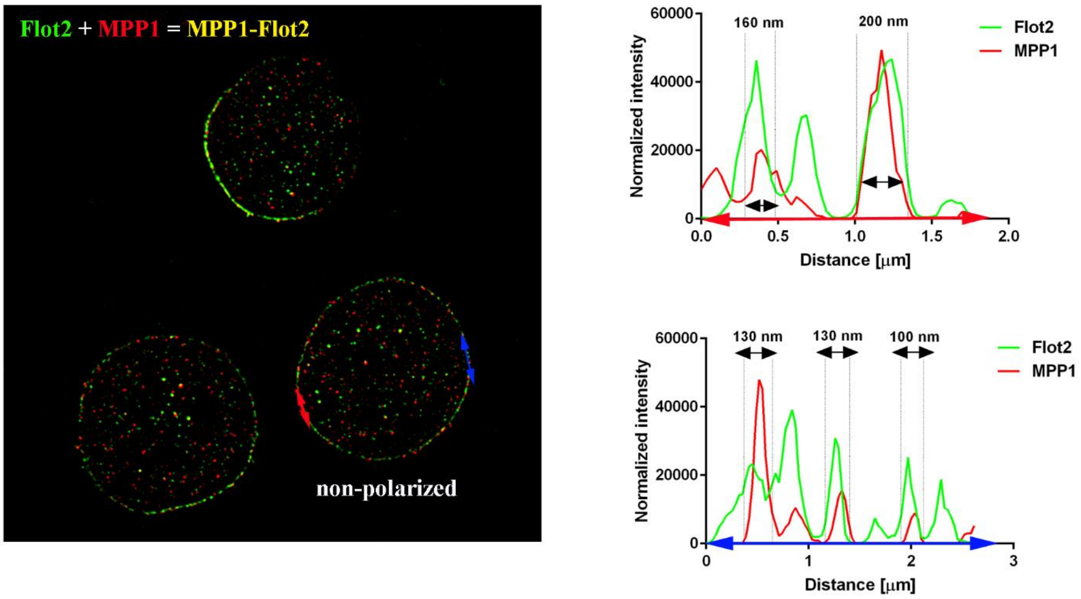

Figure S3. Size measurements of the MPP1-flotillin 2 complexes in HEL cells. (a,b) SIM^2 images (left) and fluorescence intensity profiles (right) of both flotillin 2 (Flot2) and MPP1 in selected area on the PM (red and blue arrows) in flotillin 2 polarized (a) and flotillin 2 non-

polarized cells (b). Measurements were performed using Zen 2012 BLACK software (Carl Zeiss).

Supplementary Figure 4

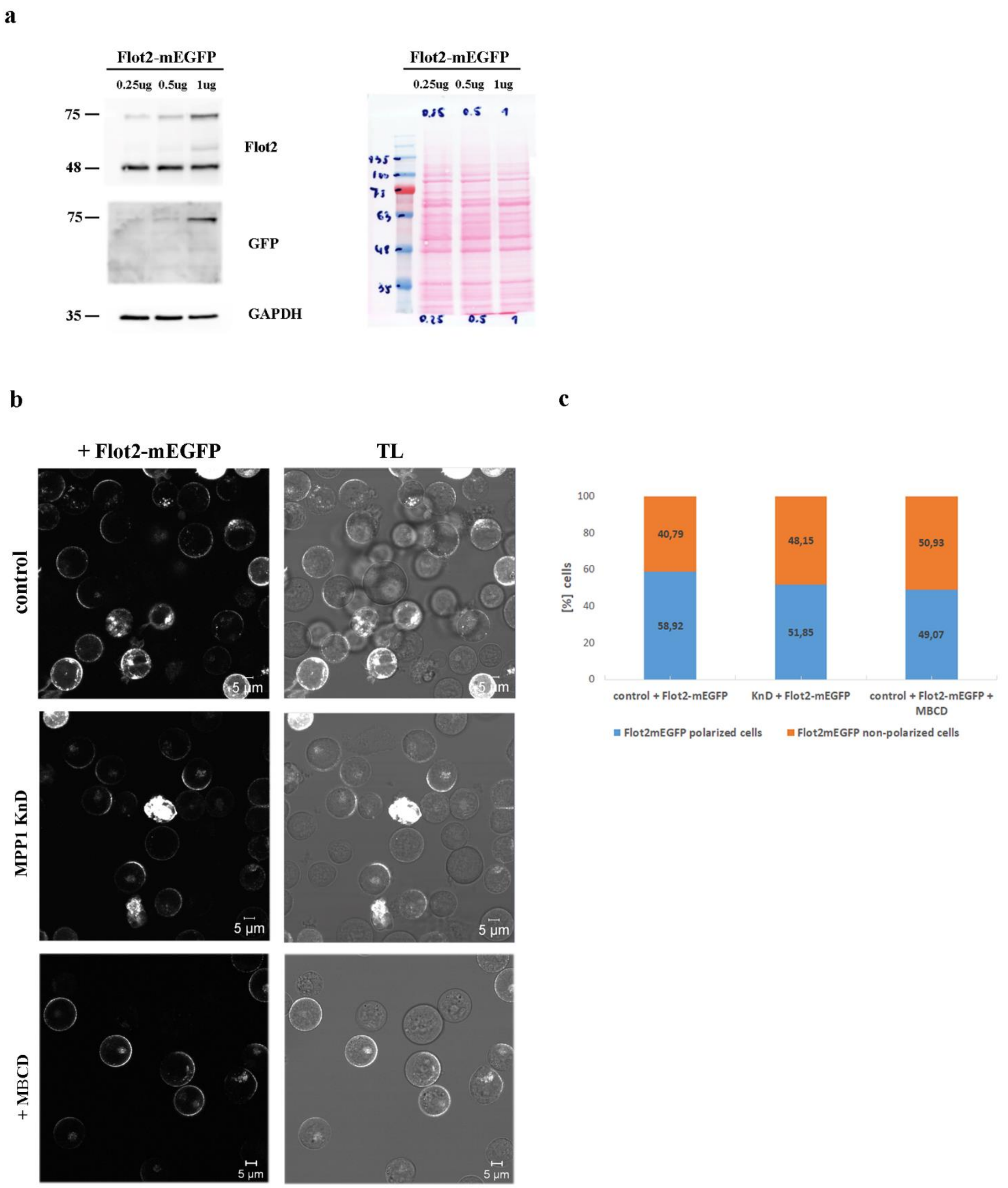

Figure S4. Analysis of overexpression level of Flot2-mEGFP in HEL cells.

(**a**) Optimization of the overexpression of Flot2-mEGFP in HEL cells. HEL cells were transfected with different amounts of the Flot2-mEGFP construct (0.25, 0.5, 1 µg DNA/ 1 million cells) and 24 h after transfection lysates were analyzed in Western blot. Both

anti-flotillin 2 (Santa Cruz sc28320) and anti-GFP (Santa Cruz; sc9996) antibodies were used to distinguish the endogenous and overexpressed level of flotillin 2. Anti-GAPDH antibodies (Santa Cruz; sc32233) and Ponceau staining (right) were used as loading controls. **(b)** Visualization of the organization of overexpressed Flot2-mEGFP (0.5 µg/24 h) in control, MPP1 KnD and MßCD treated cells. Images were obtained using a Zeiss LSM510 confocal scanning microscope (Jena, Germany) and were acquired with a 63×, 1.2NA water-immersion objective using a 488 nm argon laser as the excitation source. Scale bar, 5 µm. **(c)** Average percentage of Flo2-mEGFP polarized and Flot2-mEGFP-non-polarized cells observed in the control MPP1 KnD and MßCD treated cells after transfection.

Supplementary Figure 5

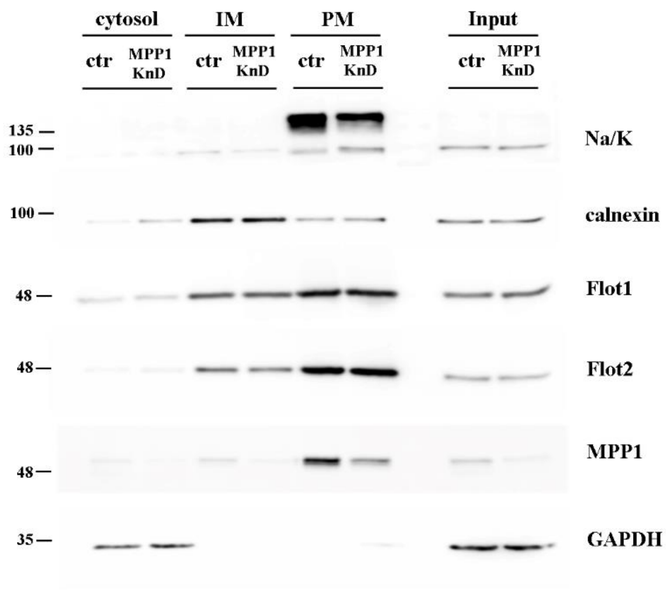

Figure S5. Analysis of distribution of flotillins in the PM and internal membranes. Fractionation of control (ctr) and MPP1 KnD cells was performed using a Plasma Membrane Extraction kit (Abcam; ab65400) according to the manufacturer's protocol. The input fractions (right) and fractions after fractionation (cytosol, internal membranes IM, plasma membrane PM) were analyzed by Western blot. Anti-calnexin (Santa Cruz; sc46669), anti-Na/K (Santa Cruz; sc48345) and anti-GAPDH antibodies were used as a marker of IM, PM and cytosol, respectively. The total level of the endogenous flotillin 1 (probed with Santa Cruz; sc133153) and flotillin 2 (probed with Santa Cruz; sc28320) in control and MPP1 KnD cells is comparable (Input). No differences in the redistribution of flotillins between PM and IM were observed in control and MPP1 KnD cells.

Supplementary Figure 6

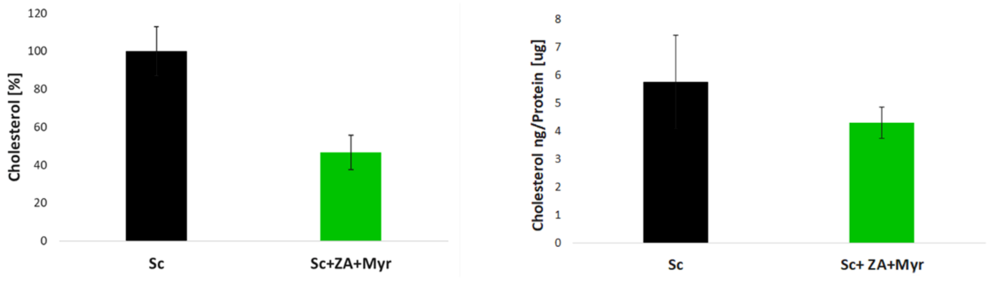

Figure S6. The effect of metabolic treatment of ZA and Myr on the total cholesterol level in HEL cells.

The total level of cholesterol in control and ZA/Myr treated cell lysates was determined with Amplex Red Cholesterol Kit Assay (Thermo Fisher).

Supplementary Figure 7

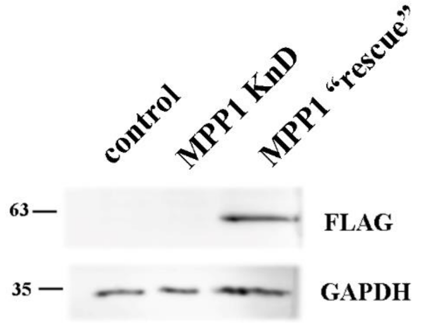

Figure S7. Restoration of MPP1 using MPP1 "rescue" overexpression in MPP1 KnD cells. Western blot analysis showing the restoration of MPP1 "rescue" expression (anti-Flag antibodies; Sigma a2220) in MPP1 KnD cells. Lysates were analyzed 48 h after transfection. Anti-GAPDH antibodies was used as a loading control.
